# Cut your losses: self-amputation of injured limbs increases survival

**DOI:** 10.1093/beheco/arx063

**Published:** 2017-04-22

**Authors:** Zachary Emberts, Christine W Miller, Daniel Kiehl, Colette M St. Mary

**Affiliations:** a Department of Biology, University of Florida, 876 Newell Drive Gainesville, FL 32611, USA,; b Entomology and Nematology Department, University of Florida, 1881 Natural Area Drive Gainesville, FL 32611, USA

**Keywords:** autotomy, Coreidae, Hemiptera, injury, natural selection, regeneration

## Abstract

Autotomy, self-induced limb loss, is an extreme trait observed throughout the animal kingdom; lizards drop their tails, crickets release their legs, and crabs drop their claws. These repeated evolutionary origins suggest that autotomy is adaptive. Yet, we do not have a firm understanding of the selective pressures that promote and maintain this extreme trait. Although multiple adaptive hypotheses exist, research has generally focused on autotomy’s adaptive value as a form of predator escape. However, autotomy could also be selected to reduce the cost of an injured limb, which we investigate here. Previously, this alternative hypothesis has been challenging to directly test because when an injury occurs on an autotomizable limb, that limb is almost always dropped (i.e., autotomy is behaviorally fixed within populations). Recently, however, we have identified a species, *Narnia femorata* (Insecta: Hemiptera: Coreidae), where some individuals autotomize limbs in response to injury, but some do not. This natural variation allowed us to investigate both the survival costs of retaining an injured limb and the benefits of autotomizing it. In this study, we find a positive association between autotomizing injured limbs and survival, thereby quantifying a new and likely widespread benefit of autotomy—reducing the cost of injury.

## Lay Summary

Here, we show that one species of leaf-footed bug can self-amputate an injured limb to reduce the cost of injury. Other benefits of self-amputation include escaping predation and escaping entrapment. By identifying different benefits of this behavior, we stand to gain a more comprehensive understanding of how such an extreme trait evolves.

## INTRODUCTION

Sacrificing a limb by self-amputation (i.e., autotomy) has evolved throughout the animal kingdom despite the enormous costs associated with this behavior (Table 1). Minimally, the cost of self-amputation includes the potential loss of blood or comparable bodily fluid (Lawrence 1992; Foelix 1996; Wilkie 2001; Fleming et al. 2007) and the potential for infection (Fleming et al. 2007; Slos et al. 2009; Bely and Nyberg 2010). Additionally, autotomy comes with costs that coincide with the function of the lost limb (Maginnis 2006; Fleming et al. 2007; Tsurui et al. 2014; Domínguez et al. 2016). These costs can be especially substantial should they decrease an individual’s future reproductive success (Smith 1992; Maginnis 2006; Fleming et al. 2007). Yet, given autotomy’s evolutionary persistence (McVean 1982; Zani 1996; Fleming et al. 2007), the benefits must outweigh the substantial costs (Arnold 1984). Thus, to better understand how this extreme trait evolves, we must identify the adaptive benefits of self-induced limb loss.

**Table 1:** A taxonomic overview of autotomy, including anecdotal evidence of autotomy in response to injury

Phyla	Group	Autotomizable appendage	Autotomy to escape	Autotomy in response to injury	Citations
Coelenterata	Jellyfish	Tentacles	Yes	—	Bickell-Page & Mackie 1991
Mollusca	Nudibranchs	Cerata	Yes	—	Marín & Ros 2004
	Bivalves	Tentacles	Yes	—	Donovan et al 2004
	Squid	Tentacles	Yes	—	Bush 2012
Annelida	Earthworms	Tail	Yes	—	Fiore et al 2004
Arthropoda	Spiders	Legs, pedipalps	Yes	Yes	Savory 1928, Punzo 1997
	Scorpions	Tail	Yes	—	Mattoni et al 2015
	Crabs	Claws, legs	Yes	Yes	McVean 1973, McVean 1982
	Centipedes	Legs	Yes	Yes	Lewis 1981
	Crickets	Legs	Yes	—	Bateman and Fleming 2006a
	True bugs	Legs	Yes	Yes	Luscher 1948, Emberts et al 2016
Echinodermata	Sea stars	Arms	Yes	Yes	Glynn 1982
	Brittlestars	Arms	Yes	—	Wilkie 2001
Chordata	Salamanders	Tail	Yes	Yes	Wake and Dresner 1967
	Lizards	Tail	Yes	Yes	Elwood et al 2012; Congdon et al. 1974
	Mice	Tail skin	Yes	—	Shargal et al 1999

One benefit of autotomy is its ability to help an individual escape predation. In this context, individuals use autotomy to break free from a predator’s grasp and, in some cases, to distract the predator. Predator distraction occurs when the predator spends time handling and/or consuming an autotomized limb as oppose to trying to catch the surviving individual. Post-autotomy tail movement (observed in some lizards and salamanders) exemplifies this benefit, as autotomized tails that wiggle have been shown to increase predator handling and consumption time, thereby allowing the individual more time to escape (Dial and Fitzpatrick 1983). Although the means of predator escape can vary, the ultimate benefit has been demonstrated in numerous taxa, including lizards (Congdon et al. 1974; Downes and Shine 2001), starfish (Bingham et al. 2000), decapods (Lawton 1989; Wasson et al. 2002), spiders (Punzo 1997; Brueseke et al. 2001), and crickets (Bateman and Fleming 2006a). However, it is important to recognize that autotomy has additional benefits beyond that of escaping predation.

Other benefits of autotomy include escaping nonpredatory entrapment (Foelix 1996) and reducing the cost of envenomation (Eisner and Camazine 1983; Ortego and Bowers 1996). Nonpredatory entrapment is frequently observed in arthropods who undergo a complex molting process (Robinson et al. 1991; Juanes and Smith 1995; Foelix 1996; Johnson and Jakob 1999; Maginnis 2008). During this process, limbs, especially elaborated and elongated ones, may get stuck and autotomy provides a viable option for escaping (Maginnis 2008). Moreover, there are also benefits of autotomy that are not related to survival. For instance, self-amputated limbs can be used as copulatory plugs to increase a male’s reproductive success (Knoflach and van Harten 2001; Knoflach 2002; Fromhage and Schneider 2006; Snow et al. 2006; Nessler et al. 2007; Uhl et al. 2009). Therefore, when considering the evolution of autotomy in a broader context, it is critically important to separate autotomy from the assumption that its sole adaptive function is to escape predation.

Another hypothesized benefit of autotomy, one that has gone untested, is that autotomy can limit damage associated with wounded body parts. In other words, if injury occurs on an autotomizable limb it is hypothesized that individuals can self-amputate (autotomize) the injured limb to reduce the cost of the injury. This hypothesis is largely inspired by physiological and behavioral observations. Physiologically, self-induced injuries (i.e., injuries induced through autotomy) are thought to quickly heal (Wake and Dresner 1967; Foelix 1996; Wilkie 2001). Therefore, if an externally induced injury was severe, self-amputating the injured limb may reduce the loss of blood and the chance of infection, ultimately increasing survival. Still, despite the anecdotal but taxonomically widespread observance of this behavior (Table 1), the benefit of autotomizing in response to injury has yet to be investigated.

For this behavior to be beneficial, the cost of the injury has to exceed the cost of autotomy. Thus, our first aim is to investigate whether injury has a higher survival and/or developmental cost than autotomy. Then, we experimentally investigate whether injured individuals can reduce this cost differential by autotomizing their damaged limb (i.e., reduce the cost of injury via autotomy).

## METHODS

### Study organism

**Figure 1:**
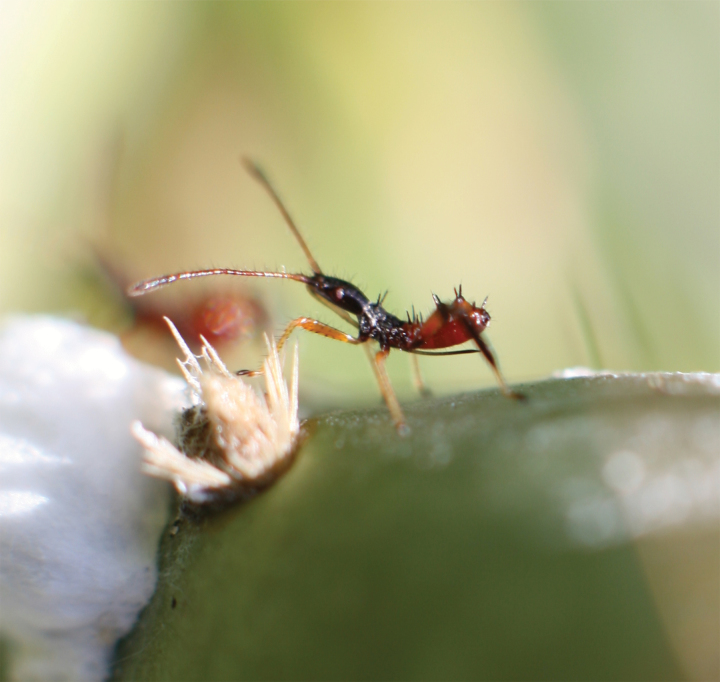
A juvenile *Narnia femorata*.

To investigate whether autotomy can indeed reduce the cost of injury, we used the leaf-footed cactus bug, *Narnia femorata* (Insecta: Hemiptera: Coreidae; Figure 1). Previously, *N. femorata* has been shown to use autotomy to escape from entrapment (Emberts et al. 2016). Furthermore, the behavior normally occurs within 60 s suggesting that individuals could also use this trait to escape from predation (Emberts et al. 2016). However, the role autotomy plays in reducing the cost of injury remains unclear.

In Hemipterans, potential responses to limb injury include autotomizing the injured limb or (retaining and) regenerating it, but not both (Luscher 1948; Shaw and Bryant 1974). In the case of autotomy, the limb is dropped at the trochanter-femur joint (Luscher 1948; Emberts et al. 2016), a location from which regeneration has not been shown to occur (Luscher 1948; Shaw and Bryant 1974). The alternative, regeneration, is only available to individuals who retain (i.e., do not autotomize) their damaged limb and have molts remaining to regrow the lost structure (i.e., juveniles). Furthermore, the regenerative capabilities are quite limited as juveniles have only been shown to partially regenerate their tibia and tarsi (Luscher 1948; Shaw and Bryant 1974). Consequently, injury location may factor into an individual’s decision to autotomize or retain an injured limb. Additionally, for *N. femorata*, the loss of a male’s hind leg may have costly implications for reproductive success, as males use their hind legs in intrasexual competition (Procter et al. 2012). Thus, our study takes sex, injury location, and the ability to regenerate into consideration.

### Study design

#### Insect rearing

For our experiments, we used first-generation lab-reared individuals. The populations were founded in November of 2015 with 29 mating pairs collected from Live Oak, Florida (30.26°N, −83.18°W). Individuals were reared in deli cups containing *Opuntia mesacantha* subsp*. lata* cladodes (cactus pads) and fruit collected from the same location throughout the experiment. Before experimentation, individuals were reared with siblings in a greenhouse (set temperature: 21–32°C, and photoperiod: 14:10 h L:D) until their second instar.

#### Experiment 1

**Figure 2:**
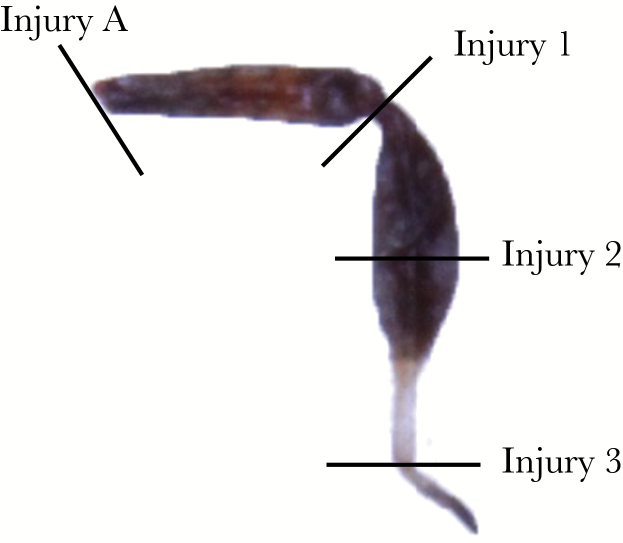
The right hind leg of a juvenile *N. femorata*, depicting the location of each injury site.

To investigate how injury and/or autotomy affects survival and development (e.g., time to reach adulthood, regeneration, and terminal body size) we randomly assigned second instar juveniles with all of their legs to one of six treatments (final sample sizes ranged from 19 to 25 per treatment): 1) control (no injury/no autotomy), 2) experimentally induced autotomy at the trochanter-femur joint, 3) incision (i.e., cut completely through the leg) at the trochanter-femur joint (henceforth referred to as Injury A to reflect that this experimentally induced injury occurred at the same location as self-induced autotomy), 4) incision at the femur-tibia joint (henceforth referred to as Injury 1), 5) incision through the middle of the tibia (henceforth referred to as Injury 2), and 6) incision at the tibia-tarsus joint (henceforth referred to as Injury 3; Figure 2). We only used individuals with all of their legs because limb loss has been shown to affect the propensity to autotomize additional limbs (Bateman and Fleming 2005). Autotomy was induced by gripping the insect’s right hind femur with reverse-action forceps while the insect was in contact with a piece of wood (38 × 44 × 305 mm; Emberts et al. 2016). For a comparative baseline, individuals in the control (no injury/no autotomy) treatment underwent a sham autotomy protocol (i.e., their legs were held for a shorter amount of time (1 s) with reverse-action forceps), but were not induced to autotomize. For the remaining treatments (e.g., injury A, 1, 2, and 3), injury was induced with iridectomy scissors at the specified location following previous regeneration protocols in other species (Luscher 1948; Shaw and Bryant 1974). After an individual’s respective procedure, it was moved into its own deli cup and placed into an incubator (temperature: 32°C, photoperiod: 14:10 h L:D). As an ethical note, animals were treated as humanely as possible by inducing injury in a disinfected environment and by providing them with species-specific optimal living conditions. Individuals were monitored daily (maximum 32 days) for developmental rate (i.e., molt timing), limb loss, and death. On becoming an adult, individuals were sexed, and their body and legs were photographed using a Canon EOS 50D digital camera attached to a Leica M165 C dissecting microscope. Pronotal width (a body size metric) and hind leg length were measured to the nearest micrometer using ImageJ v1.46.

#### Experiment 2

To experimentally test if autotomizing an injured leg increases survival, we randomly assigned juveniles (second instars) into 1 of 3 treatment groups in which the right hind leg was 1) experimentally induced to autototomize (*n* = 38), 2) injured then experimentally induced to autotomize (*n* = 39), or 3) injured without experimental autotomy (*n* = 40). This experiment involved 2 stages separated by 1 hour. First, injury was induced in treatments 2 and 3 at the femur-tibia joint with disinfected iridectomy scissors. Individuals in treatment 1 were handled in the same manner, but injury was not induced. If unplanned autotomy occurred due to handling, the individual was removed from the experiment and replaced. In the second stage (1 hour later), individuals in treatments 1 and 2 were induced to autotomize their right hind leg with reverse-action forceps, whereas individuals in treatment 3 underwent a sham autotomy protocol, as detailed above for Experiment 1. After treatment manipulations, each individual was placed into a separate deli cup with *O. mesacantha* subsp*. lata* fruit and water and moved into an incubator (temperature: 32°C, photoperiod: 14:10 h L:D). Individuals were checked at 12 h intervals over 48 h and survival and limb loss were recorded.

### Data and statistical analyses

#### Experiment 1

To investigate the effect injury has on survivorship (live/die), we conducted planned contrasts in the context of a binary, generalized linear mixed model with family as a random factor (GLMM; logit-link function assuming a binomial distribution). Since we hypothesized that injury would have a negative effect on survivorship regardless of injury site, we contrasted all of our injury treatments (injury A, 1, 2, and 3) against the control treatment (treatment 1). Using this same approach (i.e., binary GLMM using contrasts), we also investigated the effect autotomy has on survivorship by contrasting the autotomy treatment versus the control treatment. Finally, we contrasted autotomy and injuries, to evaluate whether their effects differed. Comparable analyses were done, using a GLMM with contrasts (identity-link function assuming a Gaussian distribution), to investigate whether injury and/or autotomy affected the number of days to reach adulthood or terminal body size.

To investigate whether autotomizing an injured limb resulted in higher survivorship, we compared the survival of those that autotomized their injured limbs versus those that retained them using a binary GLMM (logit-link function with assumed binomial distribution) for all 3 injury treatments combined (injury 1, 2, and 3). We excluded injury A from this and subsequent analyses because we could not determine whether individuals in this treatment autotomized their injured limb; as injury was induced at the same location where autotomy occurs. Similarly, to see whether autotomizing an injured limb affects the time to reach adulthood and/or terminal body size, we compared these metrics for individuals that retained their injured limbs versus those that autotomized their injured limb using a GLMM (identity-link function assuming a Gaussian distribution). We also investigated, using a binary GLMM (logit-link function with assumed binomial distribution), whether injury site or sex could explain any variation in the propensity to autotomize.

By using landmark locations on the legs (e.g., joints), and by measuring leg length from these landmarks, we were also able to quantify regenerative ability. If there was no growth beyond an injury site, then we classified the injury as nonregenerative.

#### Experiment 2

Our goal with the second experiment was to compare the probability of survival for those retaining injured limbs, autotomizing injured limbs, and autotomizing uninjured limbs. However, injured individuals in the “retain injured limb treatment” cannot be prevented from self-autotomizing their damaged limbs. Thus, we proceeded with a series of analyses designed to test differences across and within our treatment groups. For these tests, we used binary GLMMs (logit-link function with assumed binomial distribution) and, where relevant, used contrasts in our models to compare groups of interest for which we had developed *a priori* hypotheses of their relationships.

Across both experiments, sample sizes varied because we were unable to retrieve some individuals (*n* = 15) at the end of the experiment. Although there are multiple reasons that might explain how these individuals were lost, we believe that most of these individuals became buried in the soil on death, making it extremely challenging to locate them. Still, we took the most conservative approach and excluded these individuals from our analyses. However, even making the reasonable assumption that all 15 of these individuals died, excluding or including these individuals had no effect on our conclusions.

## RESULTS

### Experiment 1

**Figure 3:**
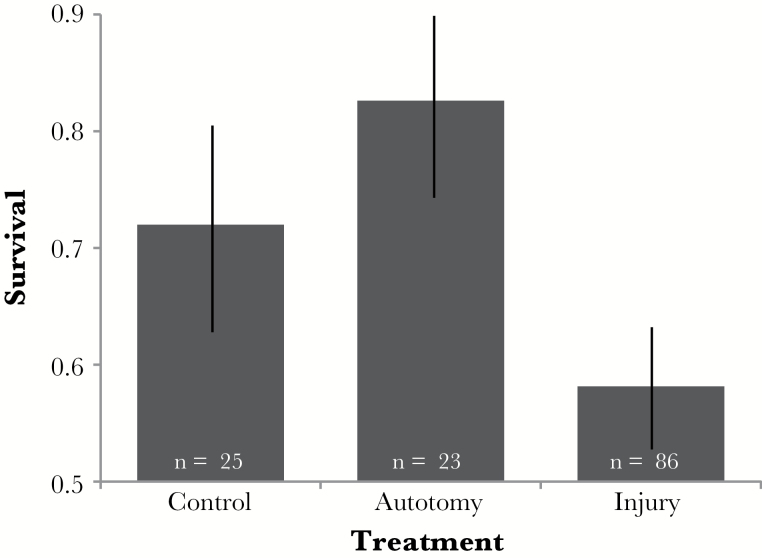
Experiment 1—contrast of treatments to investigate the effects of autotomy and injury on the proportion of individuals (±SE) surviving to adulthood.

We first compared survival for injured insects (injury locations A, 1, 2, and 3), insects experiencing experimentally induced autotomy, and individuals in our control group. We found that injured insects had approximately 25% lower survival on average than those that were experimentally induced to autotomize (GLMM with *a priori* contrasts: *χ*^2^ = 5.43, df = 1, *P* = 0.020, Figure 3). Insects that were not injured and not experimentally induced to autotomize (control group) did not differ in survival relative to the injured insects (binary GLMM with *a priori* contrasts: *χ*^2^ = 1.64, df = 1, *P* = 0.200, Figure 3) or those experiencing induced autotomy (binary GLMM with *a priori* contrasts: *χ*^2^ = 0.48, df = 1, *P* = 0.476, Figure 3). When we compared terminal body size and the number of days it took to reach adulthood across our contrasted treatments we did not find any significant differences (Table 2).

**Table 2:** Experiment 1—developmental differences between autotomy, injury, and our control (no autotomy/no injury)

	*χ* ^2^	df	*P*
Days until adulthood			
Autotomy vs. control	0.285	1	0.593
Injury vs. control	1.265	1	0.261
Autotomy vs. injury	3.37	1	0.067
Terminal body size (PW)			
Autotomy vs. control	1.2	1	0.274
Injury vs. control	0.992	1	0.319
Autotomy vs. injury	0.223	1	0.637

PW, pronotal width.

**Figure 4:**
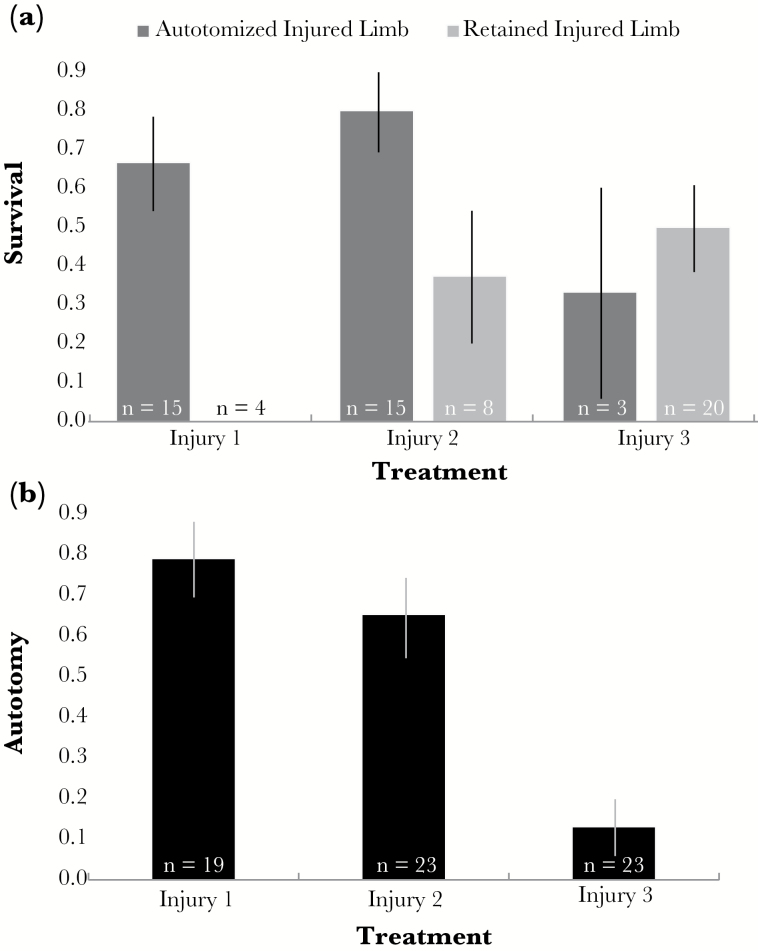
Experiment 1—effect of injury location on autotomy and survival. (a) Depicts the proportion of individuals (±SE) that survived based on their behavioral decision to autotomize or retain their injured limb for each injury location. (b) Illustrates the variation in the proportion of individuals (±SE) that autotomized at each injury location. Individuals in the injury 3 treatment had a significantly lower propensity to autotomize then those in the injury 1 and injury 2 treatments. Furthermore, autotomizing limbs injured at the injury 3 location did not increase survival.

For all of the injury treatments where autotomy was possible (injury 1, 2, and 3), a large fraction (50.7%) of individuals responded to their injury by autotomizing. In general, individuals who autotomized their injured limb had higher survival than those that retained their injured limb (binary GLMM: *χ*^2^ = 5.67, df = 1, *P* = 0.017), but the location of the injury also tended to affect this benefit (binary GLMM: *χ*^2^ = 5.53, df = 2, *P* = 0.063). In particular, autotomy of limbs injured at the femur-tibia joint (injury 1) and tibia (injury 2) led to higher survival, whereas autotomy after an injury on the tibia-tarsus joint (injury 3) did not (Figure 4).

Injury location also had an effect on the propensity to autotomize (GLMM: *χ*^2^ = 23.03, df = 2, *P* < 0.001; Figure 4). Specifically, individuals injured at the tibia-tarsus joint (injury 3) were significantly less likely to autotomize than individuals injured at the femur-tibia joint (injury 1; GLMM: *χ*^2^ = 14.79, df = 1, *P* < 0.001) and individuals injured through their tibia (injury 2; GLMM: *χ*^2^ = 20.04, df = 1, *P* < 0.001; Figure 4). Sex did not explain any of the variation in the expression of autotomy (GLMM: *χ*^2^ = 0.07, df = 1, *P* = 0.793).

Of those individuals that retained their injured limb, less than half (40.6%) survived to adulthood. Compared to those that autotomized their injured limbs, those that retained them required fewer days to reach adulthood (Table 3) and showed some form of regeneration. Individuals who retained their injured limb in the injury 3 treatment regenerated their first tarsal segment, whereas individuals in the injury 2 treatment regenerated both their tibia and their first tarsal segment. The regenerated tarsi in both treatments were 55% shorter than our control (Supplementary Table S1). Thus, we conclude that *N. femorata* has partial regenerative capabilities. None of the individuals that retained their injured limb in the injury 1 treatment survived to adulthood (0 out of 4); therefore, we were unable to quantify the potential for regeneration from this injury location.

**Table 3:** Experiment 1—developmental differences between self-autotomizing and retaining an injured limb

	*χ* ^2^	df	*P*
Days until adulthood			
Autotomy	9.085	1	0.003
Injury location	3.445	2	0.179
Autotomy × injury location	16.404	1	<0.001
Terminal body size (PW)			
Autotomy	3.554	1	0.059
Injury location	1.350	2	0.509
Autotomy × injury location	4.171	1	0.041

We investigated how injury location, the decision to autotomize, and their interaction affected the number of days it took a juvenile to reach adulthood and terminal body size. Means and standard errors are reported in Supplementary Table 2. PW, pronotal width.

### Experiment 2

**Figure 5:**
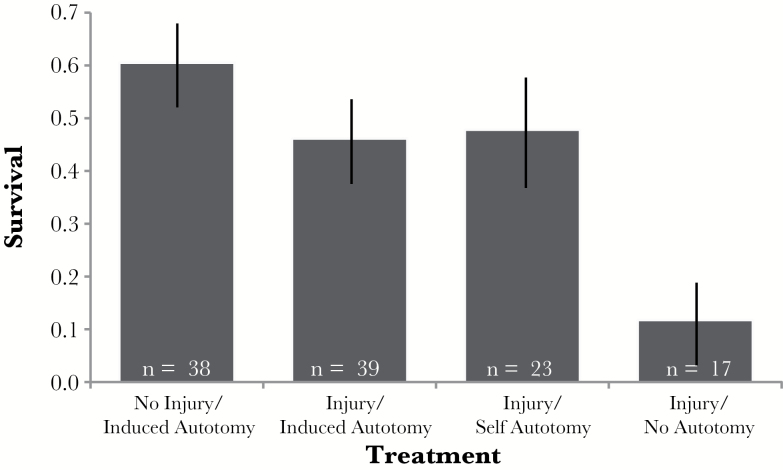
Experiment 2—proportion of individuals (±SE) that survived in each treatment based on their autotomy behavior. In treatments where autotomy was experimentally induced, individuals did not have a behavioral choice. However, when only injury was induced, an individual could have self-autotomized or retained (no autotomy) the injured limb.

Experiment 2 involved 3 treatment groups: 1) experimentally induced autotomy of a noninjured limb, 2) experimentally induced autotomy of an injured limb, and 3) injured without experimental autotomy. When we compared treatment 2 to treatment 3, we did not find that experimental autotomy of injured limbs significantly increased survival (contrasted GLMM: *χ*^2^ = 1.58, df = 1, *P* = 0.209). However, over half (57.50%) of the individuals in treatment 3 self-autotomized their injured limb. This difference in behavior allowed us to additionally consider the behavioral decision to self-autotomize an injured limb. Those that self-autotomized their injured limb had higher survivorship than those that maintained their injured limb (GLMM: *χ*^2^ = 6.10, df = 1, *P* = 0.014; Figure 5). Furthermore, those that self-autotomized their injured limbs and those that were experimentally induced to autotomize their injured limbs had similar survivorship (contrasted GLMM: *χ*^2^ < 0.001, df = 1, *P* = 0.985; Figure 5), as we hypothesized. Thus, we compared all the individuals that autotomized their injured limb, whether it was self-induced or experimentally induced, to those individuals who maintained their injured limb; we found that those who autotomized their injured limb had significantly higher survival than those who did not (contrasted GLMM: *χ*^2^ = 4.02, df = 1, *P* = 0.045; Figure 5).

## DISCUSSION

Here, we have shown that autotomy can reduce the cost of injury. Autotomy after injury has been observed across taxa (Table 1), but the benefits of the behavior have only been assumed, not tested (Savory 1928; Lewis 1981; Glynn 1982; Bulliére and Bulliére 1985; Johnson and Jakob 1999; Bingham et al. 2000; Ramsay et al. 2001). Thus, this study is the first to provide evidence of a novel benefit of autotomy—reducing the (survival) cost of injury. Other widespread benefits of autotomy include escaping predation (Congdon et al. 1974; Carlberg 1986; Lawton 1989; Punzo 1997; Bingham et al. 2000; Brueseke et al. 2001; Downes and Shine 2001; Sword 2001; Wasson et al. 2002; Bateman and Fleming 2006b) and escaping nonpredatory entrapment (Robinson et al. 1991; Juanes and Smith 1995; Foelix 1996; Johnson and Jakob 1999; Maginnis 2008). Our results, and others, highlight that there are multiple benefits of autotomy, which may select for and maintain the trait. As this evidence grows it becomes crucial that we abandon the assumption that autotomy’s sole, or even primary, adaptive benefit is escaping predation. By doing so, we stand to gain a more comprehensive understanding of how such an extreme trait evolves.

Another major implication of this study is that autotomy is less costly than injury, but only with respect to survival as we did not find injury to have an effect on the time to reach adulthood nor terminal body size. Regardless, these results highlight that autotomy and injury should not be considered synonymous. Specifically, autotomy is self-induced, or self-controlled (Fleming et al. 2007), injury. Recognizing this distinction is vital to understanding how autotomy can reduce the costs of injury. To elaborate, injuries, whether self-induced or externally induced, can result in blood loss and infection, both of which may ultimately result in death. Thus, there should be selection to minimize these effects, such as selection on an immune system (Medzhitov and Janeway 1997; Cooper and Alder 2006; Cerenius and Söderhäll 2011). However, what differentiates self-controlled injury from externally induced injury is that self-controlled injury can consistently occur at a very precise location. This consistency allows selection, over time, to potentially act on morphology to reduce the risk of infection and the loss of blood. Consequently, self-induced injury at a predetermined breakage plane may be less severe then externally induced injury. Previous studies have noted that self-induced injuries (i.e., due to autotomy) quickly seal and result in negligible amounts of blood loss (Wake and Dresner 1967; Foelix 1996; Wilkie 2001). However, the differences in blood loss and immune response between autotomy and externally induced injury have yet to be measured. Although we have not quantified these effects, the differences in survival observed in our study likely result from such differences.

The benefit of autotomizing an injured limb appears to vary by injury location. For example, we did not find a survival benefit of autotomy if the injury occurred at the tibia-tarsus joint, our most distal injury. This lack of a benefit might explain why insects experiencing injuries at that site rarely autotomized their limb. By retaining their injured limb, individuals could regenerate part of their tarsus and have a resulting hind leg that was only 19% shorter than our comparative baseline (Supplementary Table S1). This pattern highlights the likely trade-off individuals face between autotomizing an injured limb and retaining it. That is to say, when autotomizing a limb does not increase survival (i.e., the severity of the injury is minimal), few individuals should autotomize their limbs. However, when autotomy does increase survival (i.e., the injury is severe) individuals should readily drop their limb, even though it comes with the cost of permanently losing their leg.

Although injury location influenced the tendency to autotomize injured limbs, sex did not. In *N. femorata* male hind legs have been shown to function as sexually selected weapons (Procter et al. 2012; Nolen et al. 2017). Thus, the permanent loss of a male hind leg potentially comes with a larger cost than the loss of a female hind leg. In previous studies, when the costs and benefits of autotomy differ between the sexes there is often a corresponding difference in the propensity to autotomize (Fox et al. 1998; Wasson and Lyon 2005). For our study, however, there are several possible explanations for why we did not observe such differences. First, it is possible that the loss of a hind leg comes with an equal cost to males and females, as males may compensate, behaviorally (e.g., Berzins and Caldwell 1983) and/or morphologically (e.g., Simmons and Emlen 2006), for the loss of their weapon, an intriguing future direction. Second, it is also possible that there was a sex difference in the propensity to autotomize (that occurred on the scale of seconds, minutes, or hours), but because of our experimental design we were unable to detect the difference. Still, even if such a difference existed, sex did not ultimately affect whether or not an individual autotomized their injured limb.

Additionally, in our study system, the consequences of autotomy and regeneration are not confounded as individuals may only autotomize *or* regenerate their injured limb, but not both. In other arthropods, autotomy (at a preformed breakage plane) often precedes regeneration. Therefore, in some instances, it can be challenging to differentiate consequences of regeneration from consequences of autotomy. One of these challenges is determining whether regeneration and/or autotomy alters developmental time. In arthropods, regeneration (preceded by autotomy) is often shown to increase the amount of time it takes to develop (Maginnis 2006). However, it is possible that this developmental delay is a consequence of autotomy, not regeneration. With our study species, we are presented with a unique opportunity to separately investigate the consequences of autotomy and (independently) the consequences of regeneration. In *N. femorata*, autotomy had no effect on the number of days it took to reach adulthood (Table 2, autotomy vs. control). However, regeneration did. When comparing those that regenerated their injured limbs (without being preceded by autotomy) to those that autotomized their injured limbs (without being followed by regeneration), we found that individuals who regenerated had shorter intermolt intervals (i.e., they developed from 3^rd^ instars to adults more quickly). In Hemipterans, and other arthropods, regeneration coincides with molting. Consequently, by decreasing intermolt intervals an individual may be able to replace its missing limb more quickly (Maginnis 2006). These results could be interpreted to mean that regeneration accelerates development in *N. femorata*. However, it is also important to note that the data set from which we drew these conclusions inherently excluded individuals that did not survive to adulthood, and thereby disproportionally excluding individuals that retained their injured limb (Figure 4a). Thus, these results could also reflect that only quickly developing individuals can retain an injured limb (with subsequent regeneration) and survive until adulthood.

Our second experiment, although it did not fully demonstrate cause and effect, provides further support that autotomy of injured limbs increases survival. As with experiment 1, we found a positive association between self-autotomizing injured limbs and survival. This result could reflect (as we have postulated) that autotomizing injured limbs increases survival. However, because this result is correlative, it could also suggest that high-quality individuals (i.e., those predisposed to higher survival) are more likely to autotomize their injured limb. Thus, in our second experiment, we induced autotomy to directly investigate these alternatives. We found that experimentally inducing autotomy had the same effect as self-induced autotomy. This similarity suggests that the patterns of survivorship we observed are not due to variation in individual quality, but instead stem from autotomy of injured limbs; thereby strongly supporting the hypothesis that autotomy can indeed reduce the cost of injury.

In conclusion, the results of this study are the first to provide evidence that autotomy can reduce the cost of injury. Specifically, here, we observed a survival difference between individuals that autotomized their injured limb and those that retained it. Furthermore, in our second experiment, we observed this survival difference just 48 h post-injury, suggesting a relatively immediate benefit to autotomizing injured limbs. However, it is also possible that autotomizing injured limbs comes with long-term benefits too. For example, if a limb is severely damaged, an individual may be able to reduce the metabolic cost of carrying around a lame limb by autotomizing it. Moreover, if the species can regenerate, autotomizing a lame limb may promote the growth of a new, functional one. Such benefits of autotomizing injured limbs are not necessarily mutually exclusive alternatives. Instead, we hypothesize that these potential benefits may additionally contribute to the selection and maintenance of autotomy. Thus, to gain a better understand how this extreme trait evolves, we must continue to identify the adaptive benefits of self-inducing limb loss.

## SUPPLEMENTARY MATERIAL

Supplementary data are available at Behavioral Ecology online.

## Supplementary Material

Supplementary_Table_S2

Supplementary_Table_S1
